# Understanding Healthcare Workers Self-Reported Practices, Knowledge and Attitude about Hand Hygiene in a Medical Setting in Rural India

**DOI:** 10.1371/journal.pone.0163347

**Published:** 2016-10-06

**Authors:** Vishal Diwan, Charlotte Gustafsson, Senia Rosales Klintz, Sudhir Chandra Joshi, Rita Joshi, Megha Sharma, Harshada Shah, Ashish Pathak, Ashok J. Tamhankar, Cecilia Stålsby Lundborg

**Affiliations:** 1 Department of Public Health Sciences, Karolinska Institutet, Stockholm, Sweden; 2 Department of Public Health and Environment, R.D. Gardi Medical College, Ujjain, India; 3 International Center for Health Research, R.D. Gardi Medical College, Ujjain, India; 4 Department of Community Medicine, R.D. Gardi Medical College, Ujjain, India; 5 Department of Microbiology, R.D. Gardi Medical College, Ujjain, India; 6 Department of Pharmacology, R.D. Gardi Medical College, Ujjain, India; 7 Department of Pediatrics, R.D. Gardi Medical College, Ujjain, India; 8 Department of Women and Children’s Health, International Maternal and Child Health, Unit, Uppsala University, Uppsala, Sweden; 9 Indian Initiative for Management of Antibiotic Resistance, Department of Environmental Medicine, R.D. Gardi Medical College, Ujjain, India; University of South Australia, AUSTRALIA

## Abstract

**Aim:**

To describe self-reported practices and assess knowledge and attitudes regarding hand hygiene among healthcare workers in a rural Indian teaching hospital.

**Setting:**

A rural teaching hospital and its associated medical and nursing colleges in the district of Ujjain, India.

**Method:**

The study population consisted of physicians, nurses, teaching staff, clinical instructors and nursing students. Self-administered questionnaires based on the World Health Organization Guidelines on Hand Hygiene in Healthcare were used.

**Results:**

Out of 489 healthcare workers, 259 participated in the study (response rate = 53%). The proportion of healthcare workers that reported to ‘always’ practice hand hygiene in the selected situations varied from 40–96% amongst categories. Reported barriers to maintaining good hand hygiene were mainly related to high workload, scarcity of resources, lack of scientific information and the perception that priority is not given to hand hygiene, either on an individual or institutional level. Previous training on the topic had a statistically significant association with self-reported practice (*p* = 0.001). Ninety three per cent of the respondents were willing to attend training on hand hygiene in the near future.

**Conclusion:**

Self-reported knowledge and adherence varied between situations, but hand hygiene practices have the potential to improve if the identified constraints could be reduced. Future training should focus on enhancing healthcare workers’ knowledge and understanding regarding the importance of persistent practice in all situations.

## Introduction

Healthcare-associated infections (HCAIs) constitute a major threat to patients’ and healthcare workers’ (HCWs) safety globally[1]. Published evidence shows that hundreds of millions of patients are affected by HCAIs annually, with a greater burden in low- and middle-income countries (LMICs) [2]. Consequences of HCAIs include: prolonged hospital stay, higher rates of morbidity and mortality, a greater financial burden on health systems, high costs for patients and their families and increased antibiotic resistance (ABR) [1]. ABR implies that infections that were previously curable with antibiotics become difficult or even impossible to treat and thus become life-threatening [3].

Pathogens are frequently transmitted from one patient to another via the hands of HCWs [4]. Thus, hand hygiene (HH) is considered one of the most important components of infection control [4–6]. HH is a general term that refers to hand cleansing actions aimed at preventing colonisation of patients, endogenous and exogenous infection of patients, colonisation of the healthcare environment and HCWs as well as infections of HCWs [1]. Practices can vary between the use of only water, water and soap, water and a medicated (antiseptic) detergent or an alcohol-based hand rub (ABHR) [1,7]. Use of ABHRs requires less time and reduces skin flora more rapidly than washing [7]. Proper implementation of HH significantly reduces the risk of cross-transmission of infections in healthcare facilities [6].

Despite the importance of HH practices, global adherence among HCWs is poor, with an average overall compliance rate less than or around 50% [8–18]. However, compliance with HH practices has been shown to increase significantly following interventional HH programmes [12, 19–23]. Barriers for adherence can be found on the individual, group and institutional levels, for example, level of knowledge and education, allocated resources, working conditions, leadership, commitment and awareness [13].

In 2009, the WHO Guidelines on Hand Hygiene in Health Care were published [1]. A user-centred design approach—‘My five moments for hand hygiene’—was developed to enable trainers, observers and HCWs to understand, remember and recognise indications for HH [1, 24].

Many studies have been carried out regarding HH knowledge and practice in hospital infection control, but data is scarce from LMICs [9], including India[6, 25]. The prevention and control of HCAIs is given low priority due to a lack of trained manpower, infrastructure and surveillance systems; overcrowded and understaffed hospitals; poor sanitation; lack of clean water; lack of legislation mandating hospital accreditation; and generally poor attitudes and compliance towards basic infection control procedures amongst healthcare providers [6]. The aim of this study was to explore self-reported practices and assess knowledge and attitude regarding HH among HCWs at a rural teaching hospital and its associated medical and nursing colleges in the district of Ujjain in Central India. Further, we wanted to i) investigate potential gaps between knowledge and reported practice and ii) identify training needs and willingness to participate in training activities.

## Methods

### Setting

The study was conducted at a 570-bedded teaching hospital and its associated medical and nursing colleges in Ujjain in the state of Madhya Pradesh, India. The health indicators of the state are among the poorest in India [26]. The study setting is located outside of Ujjain city. The majority of patients visiting the hospital come from the nearby rural areas.

### Study population and participants

This was a healthcare-setting-based study, and all HCWs and nursing students in the setting were intended to be included. The study population consisted of HCWs (physicians, nurses, clinical instructors and lecturers) at the teaching hospital and the associated medical and nursing colleges as well as undergraduate students at the nursing college. The inclusion criteria were that participants worked at the teaching hospital, were involved with teaching activities at the colleges or were students at the nursing college. A total of 489 HCWs and nursing students were eligible for inclusion in the survey.

### Data collection instruments and data collection

This study was conducted in 2010–2011. The data collection instrument was developed based on the WHO Guidelines on Hand Hygiene in Health Care [1]. Some questions were added based on expert opinions from infection control specialists from the medical college and findings from a previous qualitative study in the same setting [27]. The instruments were pre-tested for face validity before use. Two different questionnaires were constructed: one for respondents having no direct patient contact (NDPC) (S1 Appendix) and the other for respondents working with direct patient contact (DPC) (S2 Appendix). NDPC respondents were physicians working in hospital departments with no direct patient contact, lecturers and clinical instructors at the medical and nursing colleges and undergraduate nursing students at the nursing college. DPC respondents were physicians and nurses working at the teaching hospital. Both NDPC and DPC questionnaires focused on knowledge and attitudes about HH while the DPC questionnaire also contained questions regarding self-reported practices.

The self-administrated questionnaires included mainly closed-ended, multi-item questions. The questionnaires were developed in English and subsequently translated into Hindi and Malayalam. The majority of nursing staff had Malayalam as their first language, there this language was also selected. Before data collection, all participants were given the same formal introduction about the research project and the purpose of the study. The time allotted for filling out the questionnaires ranged between 60–70 minutes.

### Data analyses

Data were first entered into EpiData software (Version 3.1, EpiData Software Association, Odense, Denmark). IBM SPSS Statistics software (Versions 21.0 and 22.0,SPSS Inc., Chicago, IL, USA) was used for analysis. Frequencies and percentages were determined for the categorical and binary variables. For the continuous variables, mean, median, range and standard deviation (SD) were calculated. Respondents were stratified into four categories for analysis: physicians, physicians enrolled in postgraduate training (hereafter postgraduate residents), nursing college respondents and nurses. Pearson’s Chi-Square Exact Sig. (2-sided) tests were performed for variables where stratified groups were used. A one-way between-groups ANOVA using Bonferroni correction was performed to investigate whether there were any statistically significant differences between groups in mean age, mean years of work experience in the hospital and mean years of total work experience.

Missing data varied between items and were removed from the calculations; therefore, the number of respondents is presented for each item.

Items on self-reported practice (DPC respondents), knowledge of when HH should be performed (NDPC respondents) and the assessed risk of transmission of infectious agents (all respondents) were selected for analysis in relation to the concept of ‘My five moments for hand hygiene’ [1, 24]. This resulted in 14 items from the questionnaires. Additionally, two items were selected regarding glove use. Hence, a total of 16 items were selected for analysis. Six items were categorised under moment 1 (‘before touching a patient’), two under moment 2 (‘before clean/aseptic procedure’), three under moment 3 (‘after body fluid exposure/risk’), two under moment 4 (‘after touching a patient’) and one under moment 5 (‘after touching patient surroundings’).

A self-reported practice score in HH was calculated per participant as the sum of the answers ‘always’ and ‘sometimes’ to the 16 selected items of the DPC questionnaire. ‘Always’ was assigned the value of 1, ‘sometimes’ was assigned the value of 0.5 and a zero value was assigned to ‘never’. Responses of ‘not applicable’ were removed. Similarly, a knowledge score was constructed of selected items (n = 16) for NDPC respondents. The score was created to approximate the way in which DPC respondents reported their practice of HH and the knowledge of NDPC respondents regarding when HH should be practiced. It was also created as a parameter to compare possible differences between respondents. Kruskal–Wallis and Mann–Whitney U non-parametric ANOVAs were conducted to test the relationship between the self-reported practice/knowledge scores and study participant group, sex of study participant and whether the respondent had received formal training in HH during the last three years.

### Ethical approval

Ethical approval was granted by the Ethics Committee of the R.D. Gardi Medical College (approval number 114/2010). Informed written consent was obtained from all participants. Confidentiality was guaranteed for written and verbal information. Participation was voluntary.

## Results

Information regarding the demographic characteristics of the study participants is given in Table 1.

**Table 1:** Demographic characteristics of the study population in study setting.

	Categories of respondents				
	Physicians	Postgraduate residents	Nursing college respondents	Staff nurses	Total
N of respondents	63	55	30	111	259
Male n	46	34	8	16	104
Female n	17	21	22	95	155
Median age in years [range]	38 [26–70]	27 [23–45]	24 [20–63]	22* [19–60]	26* [19–70]
Median work experience in this hospital in years [range]	2.5 [0–11]	0.5* [0.3–7.3]	1 [0–4.8]	1.5 [0.2–17.6]	1.4* [0–17.6]
Median total work experience in years [range]	9 [0–45]	1.3* [0.3–14]	2.3 [0–38]	1.5 [0.2–28]	2* [0–45]
DPC (N)	43	39	0	111	193
NDPC (N)	20	16	30	0	66

N = number of respondents of total study population, n = number of respondents of part of study population, DPC = direct patient contact, NDPC = non-direct patient contact,

*Information is missing for one respondent in these items.

The overall response rate was 53% (259/489), and amongst the respondents, 75% (n = 259) were from DPC categories. Physicians had a significantly higher mean age (*p*<0.001), longer work experience at the study hospital (*p*<0.001) and longer total work experience (*p*<0.001) compared to the other respondents. The majority of physicians (77%; n = 62)) had additional specialisation. Postgraduate residents were medical graduates under a three-year residency at the teaching hospital. Among the nursing college faculty group, 30% (n = 30) had further specialisation. Thirty per cent (n = 30) were nursing graduates while 37% (n = 30) were undergraduate nursing students. The majority of members of the nurse group (69%; n = 108) had only 10–12 years of basic school education.

It emerged that 95% (n = 246) of respondents practiced HH ‘for self-protection against infections’, while 94% (n = 247) aimed ‘to prevent spread of infection between patients’. Eighty-one percent (n = 226) of respondents reported that they do it ‘because hands get dirty’. Of the respondents, 29% (n = 254) reported that they had received formal training in HH during the past three years. The proportion of participants that had received formal training varied between respondents: physicians (18%; n = 62), postgraduate residents (18%; n = 55), nursing college respondents (40%; n = 30) and nurses (38%; n = 107). As high as 90% (n = 258) participants thought training was needed while 93% (n = 259) reported they would like to attend training in the near future.

### Self-reported HH practices amongst DPC respondents

The majority of respondents (>50%) reported ‘always’ performing HH in all situations except ‘before any direct patient contact’ and ‘between contact with different patients’ (Table 2). However, reported practices varied among the groups (Table 3). For some items, more than 10% of physicians and postgraduate residents reported they ‘never’ practiced HH. In particular, around 15% of postgraduate residents reported they ‘never’ practiced HH ‘before any direct patient contact’, ‘before contact with patients who have known ABR organism’, ‘before injections and venepuncture’ or ‘before (any) use of gloves’.

**Table 2:** Direct patient contact (DPC)respondents’ self-reported hand hygiene practices in study setting.

Moment	Item	Respondents per answer n (%)			Total n of respondents
		Always	Sometimes	Never	
Before touching a patient	1. Before any direct patient contact*	72 (43)	82 (49)	15 (9†)	169
	2. Before care of particularly susceptible patients	137(81)	26 (15)	7 (4)	170
	3. Before direct contact with patients with known ABR organisms	104 (64)	48 (30)	10 (6)	162
	4. Before contact with wounds	137 (82)	26(16)	4 (2)	167
	5. Between contact with different patients*	68 (40)	85 (50)	17 (10)	170
	6. Between contact with different patients in high-risk units (ICU, NICU, surgical wards etc.)	112 (75)	32 (21)	5 (3†)	149
Before clean/aseptic procedure	7. Before performing invasive procedures	139 (95)	5 (3)	3 (2)	147
	8. Before injections or venepuncture	87 (53)	59 (36)	17 (10†)	163
After body fluid exposure/risk	9. After contact with blood, body fluids, wounds, catheter sites or drainage sites	165 (93)	10 (6)	2 (1)	177
	10. After visible soiling of hands	165 (96)	7 (4)	-	172
	11. Between moving from a contaminated to a clean body site of the same patient*	106 (63)	48(29)	14 (8)	168
After touching a patient	12. After contact with patient’s intact skin*	112 (65)	50 (29)	10 (6)	172
	13. After contact with infectious patients	168 (95)	9 (5)	-	177
After touching patient’s surroundings	14. After contact with inanimate objects in the immediate vicinity of the patient*	89 (54)	64 (39)	11 (7)	164
Glove use	15. Before using (any) gloves*	100 (58)	53 (31)	18 (11)	171
	16. After glove removal*	150 (86)	22 (13)	3 (2†)	175

n = number, % = row percentage,

* = a statistically significant difference in responses across respondent groups (*p*<0.05),

^†^ = sum of the percentages exceeds or falls below 100% due to rounding

**Table 3:** Self-reported hand hygiene practice according to respondent study groups (DPC respondents) in study setting.

Item	Respondents per answer n (%)			χ²
	Physicians	Postgraduate residents	Staff Nurses	*p*-value†
Before any direct patient contact	n = 40	n = 39	n = 90	<0.001
Always	12 (30)	9 (23)	51 (57)	
Sometimes	22 (55)	23 (59)	37 (41)	
Never	6 (15)	7 (18)	2 (2)	
Between contact with different patients	n = 40	n = 39	n = 91	0.008
Always	9 (23)	11 (28)	48 (53)	
Sometimes	26 (65)	23 (59)	36 (40)	
Never	5 (13*)	5 (13)	7 (8*)	
Between moving from a contaminated to a clean body site of the same patient	n = 38	n = 39	n = 91	<0.001
Always	24 (63)	14 (36)	68 (75)	
Sometimes	9 (24)	21 (54)	18 (20)	
Never	5 (13)	4 (10)	5 (5)	
After contact with patient’s intact skin	n = 41	n = 37	n = 94	<0.001
Always	15 (37)	15 (41)	82 (87)	
Sometimes	20 (49)	18 (49)	12 (13)	
Never	6 (15*)	4 (11*)	-	
After contact with inanimate objects in the immediate vicinity of the patient	n = 37	n = 37	n = 90	0.004
Always	13 (35)	16 (43)	60 (67)	
Sometimes	19 (51)	17 (46)	28 (31)	
Never	5 (14)	4 (11)	2 (2)	
Before using (any) gloves	n = 42	n = 38	n = 91	0.017
Always	32 (76)	15 (39)	53 (58)	
Sometimes	7 (17)	16 (42)	30 (33)	
Never	3 (7)	7 (18*)	8 (9)	
After glove removal	n = 41	n = 38	n = 96	0.017
Always	30 (73)	30 (79)	90 (94)	
Sometimes	10 (24)	7 (18)	5 (5)	
Never	1 (2*)	1 (3)	1 (1)	

n = total number of respondents per group of respondents, % = column percentage

* = sum of percentages exceeds or falls below 100 due to rounding

^†^ = *p*-value shows a statistical difference in responses across groups of respondents according to Pearson’s Chi-Square Exact Sig. (2-sided) test.

The median score of the self-reported HH practice score was 12.5, and the mean was 12 out of a maximum of 16 (n = 186). Respondents who reported they had received formal training in HH in the last three years had an HH practice score statistically significantly higher (*p* = 0.001) than respondents who had not. However, no statistical significance was found when the practice score was tested for association with the group or sex of study participants.

### Knowledge amongst NDPC respondents regarding when HH should be practiced

The overall percentage of NDPC respondents who reported HH should ‘always’ be practiced varied between 77–98% amongst the various items. In regard to ‘after contact with patient’s intact skin’, 97% of nursing college respondents (n = 30) reported that HH should always be performed. Similar responses were given by 68% of physicians (n = 19) and 88% of postgraduate residents (n = 16).

The median HH knowledge score was 15and the mean was 14.6 out of a maximum of 16. No statistical significance was found when the score was tested for association with respondents’ sex, group or formal HH training in the last three years.

### Assessed risk of transmission of infectious agents amongst all study participants

In regard to the assessed risk of transmission of infectious agents, respondents who assessed a ‘high risk’ varied between items from 37–94% (Table 4). In all items but two, the majority of respondents assessed the risk of transmission of infectious agents as ‘high’ rather than ‘low’(Table 5).

**Table 4:** Perceived risk of transmission of infectious agents amongst study population.

Moment	Item	Respondents per answer n (%)			Total n of respondents
		High risk	Low risk	Don’t know	
Before touching a patient	1. Before any direct patient contact*	90 (37)	134 (55)	21 (9†)	245
	2. Before care of particularly susceptible patients	202 (82)	35 (14)	9(4)	246
	3. Before direct contact with patients with known ABR organisms*	152 (64)	69 (29)	17 (7)	238
	4. Before contact with wounds	190 (78)	42 (17)	11 (5)	243
	5. Between contact with different patients*	182 (77)	45 (19)	10 (4)	237
	6. Between contact with different patients in high-risk units (ICU, NICU, surgical wards etc.)*	203 (86)	16 (7)	16 (7)	235
Before clean/aseptic procedure	7. Before performing invasive procedures*	170 (70)	54 (22)	19 (8)	243
	8. Before injections or venepuncture*	151 (63)	74 (31)	13 (5†)	238
After body fluid exposure/risk	9. After contact with blood, body fluids, wounds, catheter sites or drainage sites*	204 (84)	32 (13)	7 (3)	243
	10. After visible soiling of hands	207 (86)	29 (12)	6 (2)	242
	11. Between moving from a contaminated to a clean body site of the same patient	169 (71)	58 (24)	12 (5)	239
After touching a patient	12. After contact with patient’s intact skin*	113 (47)	115 (48)	10 (4†)	238
	13. After contact with infectious patients	227 (94)	8 (3)	6 (2†)	241
After touching patient’s surroundings	14. After contact with inanimate objects in the immediate vicinity of the patient	152 (64)	78 (33)	8 (3)	238
Glove use	15. Before using (any) gloves	120 (50)	100 (42)	19 (8)	239
	16. After glove removal	114 (48)	111 (46)	15 (6)	240

n = number, % = row percentage,

* = a statistically significant difference in responses between groups of respondents (*p*<0.05),

^†^ = sum of the percentages exceeds or falls below 100% due to rounding

**Table 5:** Perceived risk of transmission of infectious agents according to respondent study groups in study setting.

Item	Respondents per answer n (%)				χ²
	Physicians	Postgraduate residents	Nursing college	Staff Nurses	*p*-value †
Before any direct patient contact	n = 61	n = 55	n = 30	n = 99	<0.001
High risk	28 (46)	23 (42)	21 (70)	18 (18)	
Low risk	33 (54)	30 (55)	8 (27)	63 (64)	
Don’t know	-	2 (4*)	1 (3)	18 (18)	
Before direct contact with patients with known ABR organisms	n = 59	n = 55	n = 28	n = 96	0.002
High risk	45 (76)	42 (76)	18 (64)	47 (49)	
Low risk	12 (20)	10 (18)	10 (36)	37 (39)	
Don’t know	2 (3*)	3 (5*)	-	12 (13*)	
Between contact with different patients	n = 59	n = 53	n = 27	n = 98	0.015
High risk	42 (71)	38 (72)	19 (70)	83 (85)	
Low risk	15 (25)	14 (26)	8 (30)	8 (8)	
Don’t know	2 (3*)	1 (2)		7 (7)	
Between contacts with different patients in high-risk units (ICU, NICU, surgical wards etc.)	n = 59	n = 54	n = 28	n = 94	0.001
High risk	54 (92)	51 (94)	26 (93)	72(77)	
Low risk	5 (8)	2 (4)	2 (7)	7 (7)	
Don’t know	-	1 (2)	-	15 (16)	
Before performing invasive procedures	n = 61	n = 55	n = 30	n = 97	<0.001
High risk	57 (93)	48 (87)	20 (67)	45 (46)	
Low risk	4 (7)	5 (9)	9 (30)	36 (37)	
Don’t know	-	2 (4)	1 (3)	16 (16*)	
Before injections or venepuncture	n = 59	n = 55	n = 29	n = 95	<0.001
High risk	47 (80)	40 (73)	22 (76)	42 (44)	
Low risk	11 (19)	12 (22)	7 (24)	44 (46)	
Don’t know	1 (2*)	3 (5)	-	9 (9*)	
After contact with blood, body fluids, wounds, catheter sites or drainage sites	n = 60	n = 55	n = 30	n = 98	<0.001
High risk	59 (98)	54 (98)	27 (90)	64 (65)	
Low risk	1 (2)	1 (2)	2 (7)	28 (29)	
Don’t know	-	-	1 (3)	6 (6)	
After contact with patient’s intact skin	n = 60	n = 55	n = 29	n = 94	0.001
High risk	22 (37)	20 (36)	25 (86)	46 (49)	
Low risk	35 (58)	33 (60)	4 (14)	43 (46)	
Don’t know	3 (5)	2 (4)	-	5 (5)	

n = total number of respondents per group of respondents,% = row percentage

* = sum of percentages exceeds or falls below 100% due to rounding

^†^ = *p*-value shows a statistical difference in responses across groups of respondents according to Pearson’s Chi-Square Exact Sig. (2-sided) test.

### Barriers for non-compliance with HH practice

The main reported barriers contributing to non-compliance with HH practices amongst all respondents are presented in Table 6.

**Table 6:** Perceived main barriers for non-compliance with hand hygiene amongst study population.

Barrier	n (%)
1. Lack of time (overburdened by work)	239 (73)
2. Irregular water supply	236 (71)
3. Emergency in workplace	237 (70)
4. No facility for hand washing	233 (65)
5. Inaccessible hand washing supplies	235 (63)
6. Lack of active participation in hand hygiene promotion at individual or institutional level	232 (49)
7. Lack of institutional safety climate/culture of personal accountability of HCWs	229 (48)
8. Lack of institutional priority for hand hygiene	229 (47)
9. Lack of scientific information regarding definitive impact of improved hand hygiene on HCAI rates	231 (44)
10. Lack of administrative sanction of non-compliers or rewarding of compliers to perform hand hygiene	231 (42)
11. Absence of hand washing guidelines in hospital	232 (40)
12. Lack of role model from colleagues or superiors	234 (34)

n = number; HCW = healthcare worker; HCAI = healthcare-associated infection

### Materials used for washing/drying of hands and self-reported frequency of HH practices

In terms of materials used for HH practices, ‘Water and soap cake/detergent cake’ was found to be the most common material used in all given work places (63–82%), followed by ‘Spirit/ABHR’, with the highest percentage in the operation theatre (OT), where just over half of all respondents reported using it (54%; n = 74). Optional materials included ‘Only water’, ‘Water and antiseptic solution’ and ‘Water and liquid soap’. The most common reported practice for drying hands after washing was found to be ‘Own handkerchief’ (57%; n = 144) followed by ‘Air dry’ (48%; n = 140).

## Discussion

To our knowledge, this is one of the few studies in India exploring the self-reported practices, knowledge and attitude regarding HH amongst HCWs, medical teaching staff and nursing students [28–30]

In this study, the concept of ‘My five moments for hand hygiene’ formed a framework for identifying and exploring situations in which HH is indicated [1, 24]. The data showed that consistent self-reported compliance with HH practices as recommended by WHO [1] varied between activities and situations as well as within moments(from 40–96%). The majority of respondents reported practicing HH to protect themselves from infection. Similarly, self-protection was expressed as a primary reason for HH practice in a qualitative study of HCWs in the Netherlands [31] and in a multimodal study of nurses, mothers and children in Australia [32]. Another reason given for practicing HH in our study was ‘to prevent spread of infection between patients’, and more than 80% of respondents reported that they practiced HH ‘before care of particularly susceptible patients’, ‘before performing invasive procedures’ or ‘before contact with wounds’. In a study from Geneva, a high self-reported rate of adherence to HH was defined as performance of HH in 80% or more of all possible situations [33].

Numerous studies have shown that HH practices are employed more frequently ‘after’ rather than ‘before’ patient contact [9, 23, 34–38]. This is similar to the variation between items and moments in our study and highlights a self-protective aspect of HH practice amongst HCWs.

In relation to antibiotic use, human behaviour is influenced by complex processes including factors such as knowledge, attitudes, social norms, socio-economic conditions, peer pressure, experiences and biophysical and socio-behavioural environment [39]. The same complex process could be applicable to HH behaviour.

In a qualitative study in our setting, all participating HCWs were aware of the role their hands play in the transmission of HCAIs, and their level of knowledge on the topic was reported as relatively high. However, the participants reported low adherence to HH practices [27]. The reported barriers were in accordance with the findings of the present study, in which the five most common barriers reported by more than 60% of the respondents were connected to lack of time and scarcity of resources. In a recent study amongst physicians at a tertiary care hospital in West Bengal, patient load and material shortages were reported as the major barriers to HH practices [40]. This is consistent with findings from another LMIC (Pakistan), where a lack of materials was the most commonly reported barrier to HH compliance amongst trainee physicians [9]. Similar to the most commonly reported barrier in our study, observational studies of HCWs in various wards in a European setting and in ICUs in India have demonstrated that highly intense patient care is associated with low compliance with HH practices [8, 15]. The same has been reported for ICU nurses in India [41]; however, the opposite has also been reported [9, 19]. Sax et al. [33] found that the perception that HH is relatively easy to perform was independently associated with high self-reported practice amongst HCWs in Switzerland.

In an observational study of nursing students in the USA, it was discovered that the HH practices of a student’s mentor were the strongest predictor of the student’s practices; a student was 70% more likely to engage in HH if the mentor did so[42]. A majority of medical students in Saudi Arabia reported that the behaviour of teachers was the most important factor for HH adherence [43]. Nurses have been found to be more sensitive to the role modelling of senior physicians and administrators than of nurse colleagues [32]. Nurses and nurse assistants have also been found to experience higher pressure from colleagues and superiors than physicians [33]. The population in our study was young, and the majority had little experience working in healthcare. This was particularly evident among postgraduate residents and staff nurses. Similar age characteristics have been reported in teaching hospitals in other LMICs [9, 44]. The largest group of participants in our study included nurses, and most had only basic school education. Physicians had a significantly higher mean age and longer work experience in this particular setting as well as more total experience compared to the other categories of respondents; hence, their experiences and behaviour are important to their younger and inexperienced colleagues. This could provide an incentive for physicians in our setting to increase their HH practices to positively influence their colleagues and students. HH adherence amongst physicians has been found to be associated with a belief that the physicians serve as role models to colleagues [45], and in an earlier study from India, the majority of physicians thought that their behaviour influenced their colleagues [40]. Another study, however, found that the majority of HCWs in ICUs did not believe that their behaviour influenced their colleagues’ behaviour, nor did most respondents believe that their colleagues adhered to HH recommendations [15].

A large majority of participants in our study claimed that they practiced HH to prevent the transmission of infectious agents; however, more than 40% of respondents pointed to a lack of scientific information demonstrating that HH prevents the spread of HCAIs. Similar disbelief in HH efficacy was expressed in a Dutch study, where physicians stated that their non-compliance was due to a lack of evidence showing that HH counteracts HCAIs [31].

Despite the fact that the majority of respondents in our study reported practicing HH to prevent the spread of infections between patients, the assessed risk of transmission varied between groups of respondents. Nurses often reported that they did not know whether a situation was connected to a ‘high’ or ‘low’ risk of transmission of infectious agents. Situations in which HCWs perceive a ‘high risk’ of transmission are likely to be connected with the perception that HH practices are mandatory. For infection risk management by infection control teams, identification, assessment and analysis of ‘risks’ or unsafe practices and their consequences are based on the potential impact (severity) of an event as well as its likelihood (frequency) [46]. Lack of knowledge, resources, commitment and understanding are aspects that could be targeted to reduce unsafe practices [46].

There was a significant positive association of the HH practice score with formal training in HH. Positive effects on HH compliance from training or exposure to campaigns have been reported in other low- and middle-income settings in Pakistan, India and Turkey [9, 16, 17, 40, 42] as well as in a high-income setting in Europe [33]. However, a follow-up study showed that HH compliance declined two years after the campaigns, implying that repeated training is required [16]. The majority of our respondents reported that they believed training was necessary and they hoped to attend such training in the future.

Nurses have the most opportunities to practice HH in their daily work [8, 15, 18], and since the majority of our staff nurses have no training beyond basic school education, HH training is important to enhance their knowledge on the topic and increase their adherence in situations requiring HH.

For some items related to self-reported practice, the majority of respondents, particularly physicians and postgraduate residents, reported that they perform HH ‘sometimes’ rather than ‘always’. Responses of ‘sometimes’ might reflect the working conditions in this setting. As noted by Sax et al. [33], self-reported practice does not measure actual adherence. Therefore, it must be understood that the figures for ‘always’ might reflect knowledge of when HH should be performed rather than actual behaviour. Additionally, self-reported practice does not provide any information on the quality of the HH practices.

### Methodological considerations

Research from LMICs on HH practice amongst HCWs is scarce. Hence, this study contributes to increasing knowledge on this topic. This kind of survey is a useful step in the overall surveillance of HCAIs. Self-reported practice, knowledge and attitudes were reported for HCWs at the rural teaching hospital, the medical college and the nursing college, providing baseline information from those working clinically with or without direct patient contact in this setting.

The study had some limitations. Self-reported practice is an inexpensive approach, but over-reporting of a desired behaviour must be considered when interpreting results [1]. Hopefully, since the respondents were granted confidentiality, the possibility of over-reporting was reduced. Self-reporting has been recognised as a limitation since it does not measure actual adherence to HH practices amongst respondents [33]. However, the results between observations and self-assessment were found to be consistent [47], which suggests self-reporting can be a good indicator of actual practice.

Missing data was found in varying proportions in answers to most questions and their corresponding items. Some respondents did not reply to questions while others chose the answer ‘not applicable’.

Even though all HCWs and nursing students were intended to be included in the study population, due primarily to logistical difficulties, the response rate was 53%. It is possible that the participants in this study might have a higher tendency to perform HH or simply find the topic more interesting and important than the non-participating HCWs. The respondents may also be habitual survey responders; therefore, responder bias is possible. Numerical generalizability from this study should not really be done. However, we do consider these hospitals to be relatively representative for many hospitals in India and thus consider the results to be to some extent generalizable in a more qualitative sense, i.e. as we find these results in our study, the situation is likely relatively similar in many other institutions as well.

## Conclusions and Recommendations

Examining the self-reported HH practices in our study setting and applying the concept of ‘My five moments for hand hygiene’, it is evident that there are gaps in understanding HH in this study setting. Even though HH is reportedly performed to a great extent in certain situations, and while knowledge of when it should be performed is high, HH is not practiced in all situations dictated by WHO guidelines. Similar to results from other studies in India and other LMICs, HCWs in this setting report being overburdened by work and that essential resources required to perform HH are scarce. To enhance HH compliance in our setting, materials must be supplied and institutional and administrative involvement needs to be increased. Future training in HH must be designed not only to influence behaviour change but also to enhance the understanding of how persistent practices in all situations have a long-term impact on reducing HCAIs and ABR. This study provides a baseline for an intervention study conducted in this teaching hospital setting and serves as a platform for future interventions and evaluations. If self-reported practice is interpreted as awareness or understanding of when HH should be practiced, and hence reflects this awareness rather than actual practice, it would also be beneficial to conduct observational studies to determine adherence to HH practices. A suggestion might be to establish patient involvement in this setting in the future. By empowering patients, they will also come to understand the necessity of HH practices and encourage their healthcare providers and visitors to acknowledge HH, contributing to reducing the cross-transmission of infectious agents in healthcare facilities.

## Supporting Information

S1 Appendix (PDF)

S2 Appendix (PDF)
