# Supplementary material for: Understanding Healthcare Workers Self-Reported Practices, Knowledge and Attitude about Hand Hygiene in a Medical Setting in Rural India
Source: PLoS One. 2016 Oct 6;11(10):e0163347. doi: 10.1371/journal.pone.0163347 (PMC5053486; doi:10.1371/journal.pone.0163347)
Supplement: S1 Appendix — (PDF) [file pone.0163347.s001.pdf]

Appendix- 1

Hand Hygiene Awareness / Practice
(for respondents having no direct patient contact (NDPC))

Date: ______________S.No_____Code No ______________

Name:	Age:	Department:
Designation:	Sex	Exp in this hospital Yrs _____ Months____
Educational Qualification	Contact No:	Total Work Exp: Yrs _____ Months____

1. **When do you think hand hygiene should be practiced at your work?**

1.	Before performing invasive procedures	⇒	Always / Some times / Never / NA
2.	Before any direct patient contact	⇒	Always / Some times / Never / NA
3.	Before beginning of work-shifts	⇒	Always / Some times / Never / NA
4.	Before care of particularly susceptible patients	⇒	Always / Some times / Never / NA
5.	Before contact with catheter sites and drainage sites	⇒	Always / Some times / Never / NA
6.	Before eating	⇒	Always / Some times / Never / NA
7.	Before contact with wounds	⇒	Always / Some times / Never / NA
8.	Before using (any) gloves	⇒	Always / Some times / Never / NA
9.	Before using sterile gloves for invasive procedures (not surgical)	⇒	Always / Some times / Never / NA
10.	Before direct contact with patients who have known antibiotic resistant organisms	⇒	Always / Some times / Never / NA
11.	Before preparing and giving medication	⇒	Always / Some times / Never / NA
12.	Before handling of sterile material	⇒	Always / Some times / Never / NA
13.	Before entering the clean part of staff changing rooms of operation areas,	⇒	Always / Some times / Never / NA
14.	Before sterilization department, or other aseptic areas	⇒	Always / Some times / Never / NA
15.	Before use of computer keyboard	⇒	Always / Some times / Never / NA
16.	Before injections or venepuncture	⇒	Always / Some times / Never / NA
17.	After contact with blood, body fluids, wounds, catheter sites or drainage sites	⇒	Always / Some times / Never / NA
18.	After visible soiling of hands	⇒	Always / Some times / Never / NA
19.	After glove removal	⇒	Always / Some times / Never / NA
20.	After using toilets	⇒	Always / Some times / Never / NA
21.	After contact with infectious patients	⇒	Always / Some times / Never / NA

22.	After contact with wounds	⇒	Always / Some times / Never / NA
23.	After contact with patient's intact skin	⇒	Always / Some times / Never / NA
24.	After end of work shift	⇒	Always / Some times / Never / NA
25.	After contact with inanimate objects in the immediate vicinity of the patient	⇒	Always / Some times / Never / NA
26.	After microbial contamination	⇒	Always / Some times / Never / NA
27.	After using computer keyboard	⇒	Always / Some times / Never / NA
28.	After contact with different patient	⇒	Always / Some times / Never / NA
29.	Between contact with different patients	⇒	Always / Some times / Never / NA
30.	Between coving from a contaminated to a clean body site of the same patient	⇒	Always / Some times / Never / NA
31.	Between different caring activities on the same patient	⇒	Always / Some times / Never / NA
32.	Between contact with different patients in high risk units (ICU, NICU, surgical wards etc)	⇒	Always / Some times / Never / NA

2**. How do you assess the risk of transmitting infectious agents in following**

1	Before performing invasive procedures	⇒	High Risk / Low risk / Don't know
2	Before any direct patient contact	⇒	High Risk / Low risk / Don't know
3	Before beginning of work-shifts	⇒	High Risk / Low risk / Don't know
4	Before care of particularly susceptible patients	⇒	High Risk / Low risk / Don't know
5	Before contact with catheter sites and drainage sites	⇒	High Risk / Low risk / Don't know
6	Before eating	⇒	High Risk / Low risk / Don't know
7	Before contact with wounds	⇒	High Risk / Low risk / Don't know
8	Before using (any) gloves	⇒	High Risk / Low risk / Don't know
9	Before using sterile gloves for invasive procedures (not surgical)	⇒	High Risk / Low risk / Don't know
10	Before direct contact with patients who have known antibiotic resistant organisms	⇒	High Risk / Low risk / Don't know
11	Before preparing and giving medication	⇒	High Risk / Low risk / Don't know
12	Before handling of sterile material	⇒	High Risk / Low risk / Don't know
13	Before entering the clean part of staff changing rooms of operation areas,	⇒	High Risk / Low risk / Don't know
14	Before sterilization department, or other aseptic areas	⇒	High Risk / Low risk / Don't know
15	Before use of computer keyboard	⇒	High Risk / Low risk / Don't know
16	Before injections or venepuncture	⇒	High Risk / Low risk / Don't know
17	After contact with blood, body fluids, wounds, catheter sites or drainage sites	⇒	High Risk / Low risk / Don't know
18	After visible soiling of hands	⇒	High Risk / Low risk / Don't know

19	After glove removal	⇒	High Risk / Low risk / Don't know
20	After using toilets	⇒	High Risk / Low risk / Don't know
21	After contact with infectious patients	⇒	High Risk / Low risk / Don't know
22	After contact with wounds	⇒	High Risk / Low risk / Don't know
23	After contact with patient's intact skin	⇒	High Risk / Low risk / Don't know
24	After end of work shift	⇒	High Risk / Low risk / Don't know
25	After contact with inanimate objects in the immediate vicinity of the patient	⇒	High Risk / Low risk / Don't know
26	After microbial contamination	⇒	High Risk / Low risk / Don't know
27	After using computer keyboard	⇒	High Risk / Low risk / Don't know
28	After contact with different patient	⇒	High Risk / Low risk / Don't know
29	Between contact with different patients	⇒	High Risk / Low risk / Don't know
30	Between coving from a contaminated to a clean body site of the same patient	⇒	High Risk / Low risk / Don't know
31	Between different caring activities on the same patient	⇒	High Risk / Low risk / Don't know
32	Between contact with different patients in high risk units (ICU, NICU, surgical wards etc)	⇒	High Risk / Low risk / Don't know

3. **Why do you wash your hands at your work place?**

**1**	Because hands get dirty	⇒	YES / NO / Don't Know
**2**	For self protection against infections	⇒	YES / NO / Don't Know
**3**	To prevent spread of infection between patients	⇒	YES / NO / Don't Know
**4**	Other *(Specify)*	⇒	YES / NO / Don't Know

4. What do you think the *reasons for you sometimes skipping washing hands?*

1	Lack of Time (over burdened by work)	⇒	YES / NO
2	No facility of hand washing	⇒	YES / NO
3	Due to emergency in work place	⇒	YES / NO
4	Getting late for home	⇒	YES / NO
5	Inaccessible hand washing supplies	⇒	YES / NO
6	Lack of motivation	⇒	YES / NO
7	Irregular water supply	⇒	YES / NO
8	Absence of hand washing guidelines in hospital	⇒	YES / NO
9	Hand washing agents cause irritations and dryness	⇒	YES / NO

10	Hand hygiene interferes with HCW-patient relationship	⇒	YES	/	NO
11	Skepticism about the value of hand hygiene	⇒	YES	/	NO
12	Lack of rewards/ encouragement	⇒	YES	/	NO
13	Lack of role model from colleagues or superiors	⇒	YES	/	NO
14	Lack of knowledge, experience and education	⇒	YES	/	NO
15	Not thinking about it, forgetfulness	⇒	YES	/	NO
16	Lack of administrative sanction of non-compliers or rewarding of compliers to perform hand hygiene	⇒	YES	/	NO
17	Lack of institutional safety climate/ culture of personal accountability of HCWs	⇒	YES	/	NO
18	Lack of active participation in hand hygiene promotion at individual or institutional Level	⇒	YES	/	NO
19	Lack of institutional priority for hand hygiene	⇒	YES	/	NO
20	Lack of scientific information of definitive impact of improved hand hygiene on HCAI Rates	⇒	YES	/	NO

5. In which of the following conditions you will advise your colleagues to wear gloves ?

1	Any surgical procedure	⇒	Necessary	Not Necessary
2	Conducting a vaginal delivery	⇒	Necessary	Not Necessary
3	Invasive procedures	⇒	Necessary	Not Necessary
4	Performing vascular access and procedures (central lines)	⇒	Necessary	Not Necessary
5	Contact with blood	⇒	Necessary	Not Necessary
6	Potential presence of highly infectious and dangerous organism	⇒	Necessary	Not Necessary
7	Preparing total parental nutrition	⇒	Necessary	Not necessary
8	Preparing chemotherapeutic agents	⇒	Necessary	Not necessary
9	Discontinuation of venous line	⇒	Necessary	Not Necessary
10	IV insertion and removal; drawing blood	⇒	Necessary	Not Necessary
11	Cleaning up spills of body fluids	⇒	Necessary	Not Necessary
12	Handling waste	⇒	Necessary	Not Necessary
13	Handling/cleaning instruments	⇒	Necessary	Not Necessary

Appendix- 1

14	Pelvic and vaginal examination	⇒	Necessary	Not Necessary
15	Suctioning non-closed systems of endotracheal tubes	⇒	Necessary	Not Necessary
16	Epidemic or emergency situations	⇒	Necessary	Not Necessary
17	Emptying emesis basins	⇒	Necessary	Not Necessary
18	Cleaning up spills of body fluids	⇒	Necessary	Not Necessary
19	Taking blood pressure	⇒	Necessary	Not Necessary
20	Giving oral medications	⇒	Necessary	Not Necessary
21	Distributing or collecting patient dietary trays	⇒	Necessary	Not Necessary
22	Removing and replacing linen for patient bed	⇒	Necessary	Not Necessary
23	Placing chest leads in ICU	⇒	Necessary	Not Necessary
24	Ventilation equipment and oxygen cannula	⇒	Necessary	Not Necessary
25	Using the telephone	⇒	Necessary	Not Necessary
26	Writing in the patient chart	⇒	Necessary	Not Necessary
27	Performing SC and IM injections	⇒	Necessary	Not Necessary
28	Bathing and dressing the patient	⇒	Necessary	Not Necessary
29	Caring for eyes and ears (without secretions)	⇒	Necessary	Not Necessary
30	Any vascular line manipulation in absence of blood leakage	⇒	Necessary	Not Necessary
31	Transporting patient	⇒	Necessary	Not Necessary
32	Moving patient furniture	⇒	Necessary	Not Necessary

**6. Did you receive formal training in hand hygiene in the last three years ?**	YES	NO	
**7. Do you think there is need for this kind of training /workshop?**	YES	NO	DON’T KNOW
**8. Would you like to attend such training in near future?**	YES	NO	

THANK YOU FOR YOUR PARTICIPATION
