# Supplementary material for: Understanding Healthcare Workers Self-Reported Practices, Knowledge and Attitude about Hand Hygiene in a Medical Setting in Rural India
Source: PLoS One. 2016 Oct 6;11(10):e0163347. doi: 10.1371/journal.pone.0163347 (PMC5053486; doi:10.1371/journal.pone.0163347)
Supplement: S2 Appendix — (PDF) [file pone.0163347.s002.pdf]

Appendix 2

R.D. Gardi Medical College, Ujjain
APRIAM Project
Hand Hygiene Awareness / Practice
R.D. Gardi Medical College, Ujjain
(for respondents having direct patient contact (DPC))

Date: ______________ S.No_____ Code No ______________

Name:	Age:	Department:
Designation:	Sex	Exp in this hospital Yrs _____ Months____
Educational Qualification	Contact No:	Total Work Exp: Yrs _____ Months____

**1. Encircle your work places**		**2. How often you practiced hand hygiene at your work places?**	**3. How much time do you take for washing hands?**
1	**OPD** ⇒	NEVER / 1-2 times /d / Several times /d	<1 mins / 1-3 mins / > 3-5 mins
2	**Wards** ⇒	NEVER / 1-2 times /d / Several times /d	<1 mins / 1-3 mins / > 3-5 mins
3	**ICU** ⇒	NEVER / 1-2 times /d / Several times /d	<1 mins / 1-3 mins / > 3-5 mins
4	**OT** ⇒	NEVER / 1-2 times /d / Several times /d	<1 mins / 1-3 mins / > 3-5 mins
5	**Surgical ICU** ⇒	NEVER / 1-2 times /d / Several times /d	<1 mins / 1-3 mins / > 3-5 mins
6	**PICU** ⇒	NEVER / 1-2 times /d / Several times /d	<1 mins / 1-3 mins / > 3-5 mins
7	**NICU** ⇒	NEVER / 1-2 times /d / Several times /d	<1 mins / 1-3 mins / > 3-5 mins
8	**Casualty** ⇒	NEVER / 1-2 times /d / Several times /d	<1 mins / 1-3 mins / > 3-5 mins
9	**Laboratories** ⇒	NEVER / 1-2 times /d / Several times /d	<1 mins / 1-3 mins / > 3-5 mins
10	**Blood Bank** ⇒	NEVER / 1-2 times /d / Several times /d	<1 mins / 1-3 mins / > 3-5 mins
11	**Other** ⇒	NEVER / 1-2 times /d / Several times /d	<1 mins / 1-3 mins / > 3-5 mins

4. **What material do you use for hand washing/ hand rubbing at your hospital work?**

IN OPD
- Only water
- Water & soap cake / detergent cake
- Water & antiseptic solution (savlon)
- Water and Liquid Soap
- Spirit / Alcohol based hand rub

IN WARDS
- Only water
- Water & soap cake / detergent cake
- Water & antiseptic solution (savlon)
- Water and Liquid Soap
- Spirit / Alcohol based hand rub

IN ICU
- Only water
- Water & soap cake / detergent cake
- Water & antiseptic solution (savlon)
- Water and Liquid Soap
- Spirit / Alcohol based hand rub

IN OT
- Only water
- Water & soap cake / detergent cake
- Water & antiseptic solution (savlon)
- Water and Liquid Soap
- Spirit / Alcohol based hand rub

Appendix 2

IN NICU	IN PICU
• Only water<br>• Water & soap cake / detergent cake<br>• Water & antiseptic solution (savlon)<br>• Water and Liquid Soap<br>• Spirit / Alcohol based hand rub	• Only water<br>• Water & soap cake / detergent cake<br>• Water & antiseptic solution (savlon)<br>• Water and Liquid Soap<br>• Spirit / Alcohol based hand rub
**IN CASUALTY**	**IN**________________________
• Only water<br>• Water & soap cake / detergent cake<br>• Water & antiseptic solution (savlon)<br>• Water and Liquid Soap<br>• Spirit / Alcohol based hand rub	• Only water<br>• Water & soap cake / detergent cake<br>• Water & antiseptic solution (savlon)<br>• Water and Liquid Soap<br>• Spirit / Alcohol based hand rub

5. How do you dry your hands after washing?

Clean Towel (new towel each time)	⇒	YES	NO
Used Towel	⇒	YES	NO
Own handkerchief	⇒	YES	NO
By rubbing hands on your cloths (like apron, trousers, sari etc)	⇒	YES	NO
Tissue paper	⇒	YES	NO
Air Dry	⇒	YES	NO
Autoclaved newspapers	⇒	YES	NO

6 **What areas of hands would not be cleaned properly by your technique?**

Finger tips	⇒	YES	/	NO
Thumbs	⇒	YES	/	NO
Webs between the fingers	⇒	YES	/	NO
Inner border of hands	⇒	YES	/	NO
Nails	⇒	YES	/	NO

7. **When do you practice hand hygiene at your work?**

1.	Before performing invasive procedures	⇒	Always / Some times / Never / NA
2.	Before any direct patient contact	⇒	Always / Some times / Never / NA
3.	Before beginning of work-shifts	⇒	Always / Some times / Never / NA
4.	Before care of particularly susceptible patients	⇒	Always / Some times / Never / NA
5.	Before contact with catheter sites and drainage sites	⇒	Always / Some times / Never / NA
6.	Before eating	⇒	Always / Some times / Never / NA

7.	Before contact with wounds	⇒	Always / Some times / Never / NA
8.	Before using (any) gloves	⇒	Always / Some times / Never / NA
9.	Before using sterile gloves for invasive procedures (not surgical)	⇒	Always / Some times / Never / NA
10.	Before direct contact with patients who have known antibiotic resistant organisms	⇒	Always / Some times / Never / NA
11.	Before preparing and giving medication	⇒	Always / Some times / Never / NA
12.	Before handling of sterile material	⇒	Always / Some times / Never / NA
13.	Before entering the clean part of staff changing rooms of operation areas,	⇒	Always / Some times / Never / NA
14.	Before sterilization department, or other aseptic areas	⇒	Always / Some times / Never / NA
15.	Before use of computer keyboard	⇒	Always / Some times / Never / NA
16.	Before injections or venepuncture	⇒	Always / Some times / Never / NA
17.	After contact with blood, body fluids, wounds, catheter sites or drainage sites	⇒	Always / Some times / Never / NA
18.	After visible soiling of hands	⇒	Always / Some times / Never / NA
19.	After glove removal	⇒	Always / Some times / Never / NA
20.	After using toilets	⇒	Always / Some times / Never / NA
21.	After contact with infectious patients	⇒	Always / Some times / Never / NA
22.	After contact with wounds	⇒	Always / Some times / Never / NA
23.	After contact with patient's intact skin	⇒	Always / Some times / Never / NA
24.	After end of work shift	⇒	Always / Some times / Never / NA
25.	After contact with inanimate objects in the immediate vicinity of the patient	⇒	Always / Some times / Never / NA
26.	After microbial contamination	⇒	Always / Some times / Never / NA
27.	After using computer keyboard	⇒	Always / Some times / Never / NA
28.	After contact with different patient	⇒	Always / Some times / Never / NA
29.	Between contact with different patients	⇒	Always / Some times / Never / NA
30.	Between coving from a contaminated to a clean body site of the same patient	⇒	Always / Some times / Never / NA
31.	Between different caring activities on the same patient	⇒	Always / Some times / Never / NA
32.	Between contact with different patients in high risk units (ICU, NICU, surgical wards etc)	⇒	Always / Some times / Never / NA

8. How do you assess the risk of transmitting infectious agents in following

1	Before performing invasive procedures	⇒	High Risk / Low risk / Don't know

Appendix 2

2	Before any direct patient contact	⇒	High Risk / Low risk / Don't know
3	Before beginning of work-shifts	⇒	High Risk / Low risk / Don't know
4	Before care of particularly susceptible patients	⇒	High Risk / Low risk / Don't know
5	Before contact with catheter sites and drainage sites	⇒	High Risk / Low risk / Don't know
6	Before eating	⇒	High Risk / Low risk / Don't know
7	Before contact with wounds	⇒	High Risk / Low risk / Don't know
8	Before using (any) gloves	⇒	High Risk / Low risk / Don't know
9	Before using sterile gloves for invasive procedures (not surgical)	⇒	High Risk / Low risk / Don't know
10	Before direct contact with patients who have known antibiotic resistant organisms	⇒	High Risk / Low risk / Don't know
11	Before preparing and giving medication	⇒	High Risk / Low risk / Don't know
12	Before handling of sterile material	⇒	High Risk / Low risk / Don't know
13	Before entering the clean part of staff changing rooms of operation areas,	⇒	High Risk / Low risk / Don't know
14	Before sterilization department, or other aseptic areas	⇒	High Risk / Low risk / Don't know
15	Before use of computer keyboard	⇒	High Risk / Low risk / Don't know
16	Before injections or venepuncture	⇒	High Risk / Low risk / Don't know
17	After contact with blood, body fluids, wounds, catheter sites or drainage sites	⇒	High Risk / Low risk / Don't know
18	After visible soiling of hands	⇒	High Risk / Low risk / Don't know
19	After glove removal	⇒	High Risk / Low risk / Don't know
20	After using toilets	⇒	High Risk / Low risk / Don't know
21	After contact with infectious patients	⇒	High Risk / Low risk / Don't know
22	After contact with wounds	⇒	High Risk / Low risk / Don't know
23	After contact with patient's intact skin	⇒	High Risk / Low risk / Don't know
24	After end of work shift	⇒	High Risk / Low risk / Don't know
25	After contact with inanimate objects in the immediate vicinity of the patient	⇒	High Risk / Low risk / Don't know
26	After microbial contamination	⇒	High Risk / Low risk / Don't know
27	After using computer keyboard	⇒	High Risk / Low risk / Don't know
28	After contact with different patient	⇒	High Risk / Low risk / Don't know
29	Between contact with different patients	⇒	High Risk / Low risk / Don't know
30	Between coving from a contaminated to a clean body site of the same patient	⇒	High Risk / Low risk / Don't know
31	Between different caring activities on the same patient	⇒	High Risk / Low risk / Don't know
32	Between contact with different patients in high risk units	⇒	High Risk / Low risk / Don't know

Appendix 2

(ICU, NICU, surgical wards etc)

9. **Why do you wash your hands at your work place?**

**1**	Because hands get dirty	⇒	YES	/	NO /	Don't Know
**2**	For self protection against infections	⇒	YES	/	NO /	Don't Know
**3**	To prevent spread of infection between patients	⇒	YES	/	NO /	Don't Know
**4**	Other *(Specify)*	⇒	YES	/	NO /	Don't Know

10. What do you think the *reasons for you sometimes skipping washing hands?*

1	Lack of Time (over burdened by work)	⇒	YES	/	NO
2	No facility of hand washing	⇒	YES	/	NO
3	Due to emergency in work place	⇒	YES	/	NO
4	Getting late for home	⇒	YES	/	NO
5	Inaccessible hand washing supplies	⇒	YES	/	NO
6	Lack of motivation	⇒	YES	/	NO
7	Irregular water supply	⇒	YES	/	NO
8	Absence of hand washing guidelines in hospital	⇒	YES	/	NO
9	Hand washing agents cause irritations and dryness	⇒	YES	/	NO
10	Hand hygiene interferes with HCW-patient relationship	⇒	YES	/	NO
11	Skepticism about the value of hand hygiene	⇒	YES	/	NO
12	Lack of rewards/ encouragement	⇒	YES	/	NO
13	Lack of role model from colleagues or superiors	⇒	YES	/	NO
14	Lack of knowledge, experience and education	⇒	YES	/	NO
15	Not thinking about it, forgetfulness	⇒	YES	/	NO
16	Lack of administrative sanction of non-compliers or rewarding of compliers to perform hand hygiene	⇒	YES	/	NO
17	Lack of institutional safety climate/ culture of personal accountability of HCWs	⇒	YES	/	NO
18	Lack of active participation in hand hygiene promotion at individual or institutional Level	⇒	YES	/	NO
19	Lack of institutional priority for hand hygiene	⇒	YES	/	NO

20	Lack of scientific information of definitive impact of improved hand hygiene on HCAI Rates	⇒	YES	/	NO

11. In which of the following conditions you will advise your colleagues to wear gloves ?

1	Any surgical procedure	⇒	Necessary	Not Necessary
2	Conducting a vaginal delivery	⇒	Necessary	Not Necessary
3	Invasive procedures	⇒	Necessary	Not Necessary
4	Performing vascular access and procedures (central lines)	⇒	Necessary	Not Necessary
5	Contact with blood	⇒	Necessary	Not Necessary
6	Potential presence of highly infectious and dangerous organism	⇒	Necessary	Not Necessary
7	Preparing total parental nutrition	⇒	Necessary	Not necessary
8	Preparing chemotherapeutic agents	⇒	Necessary	Not necessary
9	Discontinuation of venous line	⇒	Necessary	Not Necessary
10	IV insertion and removal; drawing blood	⇒	Necessary	Not Necessary
11	Cleaning up spills of body fluids	⇒	Necessary	Not Necessary
12	Handling waste	⇒	Necessary	Not Necessary
13	Handling/cleaning instruments	⇒	Necessary	Not Necessary
14	Pelvic and vaginal examination	⇒	Necessary	Not Necessary
15	Suctioning non-closed systems of endotracheal tubes	⇒	Necessary	Not Necessary
16	Epidemic or emergency situations	⇒	Necessary	Not Necessary
17	Emptying emesis basins	⇒	Necessary	Not Necessary
18	Cleaning up spills of body fluids	⇒	Necessary	Not Necessary
19	Taking blood pressure	⇒	Necessary	Not Necessary
20	Giving oral medications	⇒	Necessary	Not Necessary
21	Distributing or collecting patient dietary trays	⇒	Necessary	Not Necessary
22	Removing and replacing linen for patient bed	⇒	Necessary	Not Necessary
23	Placing chest leads in ICU	⇒	Necessary	Not Necessary
24	Ventilation equipment and oxygen cannula	⇒	Necessary	Not Necessary

Appendix 2

25	Using the telephone	⇒	Necessary	Not Necessary
26	Writing in the patient chart	⇒	Necessary	Not Necessary
27	Performing SC and IM injections	⇒	Necessary	Not Necessary
28	Bathing and dressing the patient	⇒	Necessary	Not Necessary
29	Caring for eyes and ears (without secretions)	⇒	Necessary	Not Necessary
30	Any vascular line manipulation in absence of blood leakage	⇒	Necessary	Not Necessary
31	Transporting patient	⇒	Necessary	Not Necessary
32	Moving patient furniture	⇒	Necessary	Not Necessary

**12. Did you receive formal training in hand hygiene in the last three years ?**	YES	NO	
**13. Do you think there is need for this kind of training /workshop?**	YES	NO	DON'T KNOW
**14. Would you like to attend such training in near future?**	YES	NO	

THANK YOU FOR YOUR PARTICIPATION
